# Unique circulating microRNAs in relation to EGFR mutation status in Japanese smoker male with lung adenocarcinoma

**DOI:** 10.18632/oncotarget.21425

**Published:** 2017-09-30

**Authors:** Sachio Ito, Yoshihiro Kamoto, Akiko Sakai, Kaori Sasai, Tatsuro Hayashi, Shinichi Toyooka, Hiroshi Katayama

**Affiliations:** ^1^ Department of Molecular Oncology, Graduate School of Medicine, Dentistry and Pharmaceutical Sciences, Okayama University, Okayama, Japan; ^2^ Division of Thoracic Surgery, National Hospital Organization, Yamaguchi-Ube Medical Center, Yamaguchi, Japan; ^3^ Department of Thoracic, Breast and Endocrinological Surgery, Graduate School of Medicine, Dentistry and Pharmaceutical Sciences, Okayama University, Okayama, Japan; ^4^ Department of Clinical Genomic Medicine, Graduate School of Medicine, Dentistry and Pharmaceutical Sciences, Okayama University, Okayama, Japan

**Keywords:** circulating miRNA, lung adenocarcinoma, EGFR gene mutation, smoking, male

## Abstract

The incidence of lung adenocarcinoma has been increasing recently in smokers. The molecular target therapy has been developed for lung adenocarcinoma patients harboring EGFR gene mutation. However, the treatment modalities for patients without mutation are currently limited. Thus, analysis of EGFR gene mutation status at early stage is important strategy to classify the patients for improving treatments and prognosis efficiently. This study aimed to identify microRNA (miRNA) signature in relation to mutation status in EGFR gene in early stage of lung adenocarcinoma male patients with smoking history. MiRNA profiles were assessed by microarray in paired plasma and tissue pooled from 10 EGFR wild type (EGFR-wt) and 10 EGFR mutated (EGFR-mut) patients. Expressions of selected miRNAs were verified further by real-time qRT-PCR in 83 plasma samples consisting of 55 EGFR-wt patients and 28 EGFR-mut patients and their correlation with clinicopathological parameters and EGFR gene mutation status were evaluated. We found that seven miRNAs (miR-16-5p, miR-23a-3p, miR-103a-3p, miR122-5p, miR-223-3p, miR-346 and miR-451a) were differentially expressed in stage I and stage I+II. Especially, miR-23a-3p was only miRNA shown higher expression in EGFR-wt patients than EGFR-mut patients. Thus, our findings could be useful non-invasive biomarkers to differentiate mutation status in EGFR gene in smoker lung adenocarcinoma male patients.

## INTRODUCTION

Lung cancer is one of the most lethal malignancies and the most common cause of cancer-related death [1]. Non-small cell lung cancer (NSCLC), which accounts of about 85% of all lung cancers, is histopathologically classified into adenocarcinoma, squamous cell carcinoma, and large cell carcinoma. Incidence of squamous cell carcinoma used to be the predominant form of NSCLC, however, it has been replaced by adenocarcinoma since last few decades [2, 3]. Somatic mutations in the epidermal growth factor receptor (EGFR) gene occur in more than half cases of lung adenocarcinoma in Japan [4] and those are associated with good responsiveness to EGFR tyrosine kinase inhibitors (TKIs), gefitinib and erlotinib [5, 6].

Interestingly, epidemiological studies have revealed that EGFR gene mutations are more common in female than male and occur significantly to non-smokers rather than smokers [4]. Cigarette smoking is well established risk factor and is a significant contributor to morbidity and mortality for lung cancer. Our previous epidemiological study has shown that Japanese males with smoking history has about 7.9 times risk to develop lung adenocarcinoma in EGFR-wt males (unpublished data). Therefore, the comprehensive studies on genetic alterations such as driver and passenger gene mutations associated with smoking in male patients with lung adenocarcinoma have been extensively conducted last decades. However, identification of genetic mutation for early detection and molecular target treatment of smoker male adenocarcinoma patients remain to be elucidated.

MicroRNAs (miRNAs), small non-cording RNAs with 18-25 nucleotides in length that negatively regulate mRNA expression through direct inhibition of translation or induction of mRNA degradation, are key contributors to smoking response, tumorigenesis and treatment response and exert a wide range of biological function [7–10]. Because of their prolonged stability in bloodstream and relatively easy to detect, several studies have suggested that serum and plasma miRNA (hereafter referred to as circulating miRNAs) have great potential benefit in clinical application as non-invasive biomarkers for disease detection, diagnosis and prognosis, and susceptibility to molecular targeted therapy [11–17].

Previous studies have identified several unique miRNA expression profiles related to EGFR mutational status and sensitivity to EGFR TKIs in lung adenocarcinoma tissues [18, 19]. Some type of miRNAs differentially expressed in wt or mut EGFR expressing tumor tissues have been shown significant association with smoking history, and the results, however, are inconclusive among studies probably due to possible confounding effects in patient cohorts investigated [20, 21]. Of note, several studies have reported that the expression patterns of miRNAs in tissues are significantly different from those in bloodstream in the presence or absence of smoking history [15, 22]. For example, miR-20a, miR-233, miR-21 and miR-145 are upregulated and let-7i-3p and miR-154-5p are downregulated in serum sample from smokers with lung adenocarcinoma [12, 23, 24]. Despite the accumulating evidence on circulating miRNA expression profiles related to EGFR mutational status as similar to the cases of tissues samples, there are still limited studies regarding the association between smoking history related miRNA signatures and EGFR mutational status. Recently, it has been shown that circulating miRNA-122 and miRNA-195 have prognostic value in predicting EGFR mutation and overall survival of female non-smokers with advanced stage lung adenocarcinoma [25]. Nevertheless, little is known about smoking history associated circulating miRNAs which can predict EGFR mutational status and prognosis of smoker males with lung adenocarcinoma. Smoking has a strong causal effect on the generation of lung adenocarcinoma unaccompanied EGFR mutation in males, therefore, identification of EGFR mutational status distinguishable specific miRNAs in male smokers would be effective biomarker for early diagnose of patients given either TKI therapy or conventional chemotherapy.

Here, we conducted an explorative miRNA expression study in plasma and surgically resected tumor tissues from smoker males with lung adenocarcinoma harboring wild type and mutated EGFR gene using miRNA microarray and qRT-PCR, and found a group of miRNAs correlating with the EGFR mutational status, especially in early stage of male smoker patients.

## RESULTS

### MiRNA expression profiling for selecting candidates

**Figure 1:**
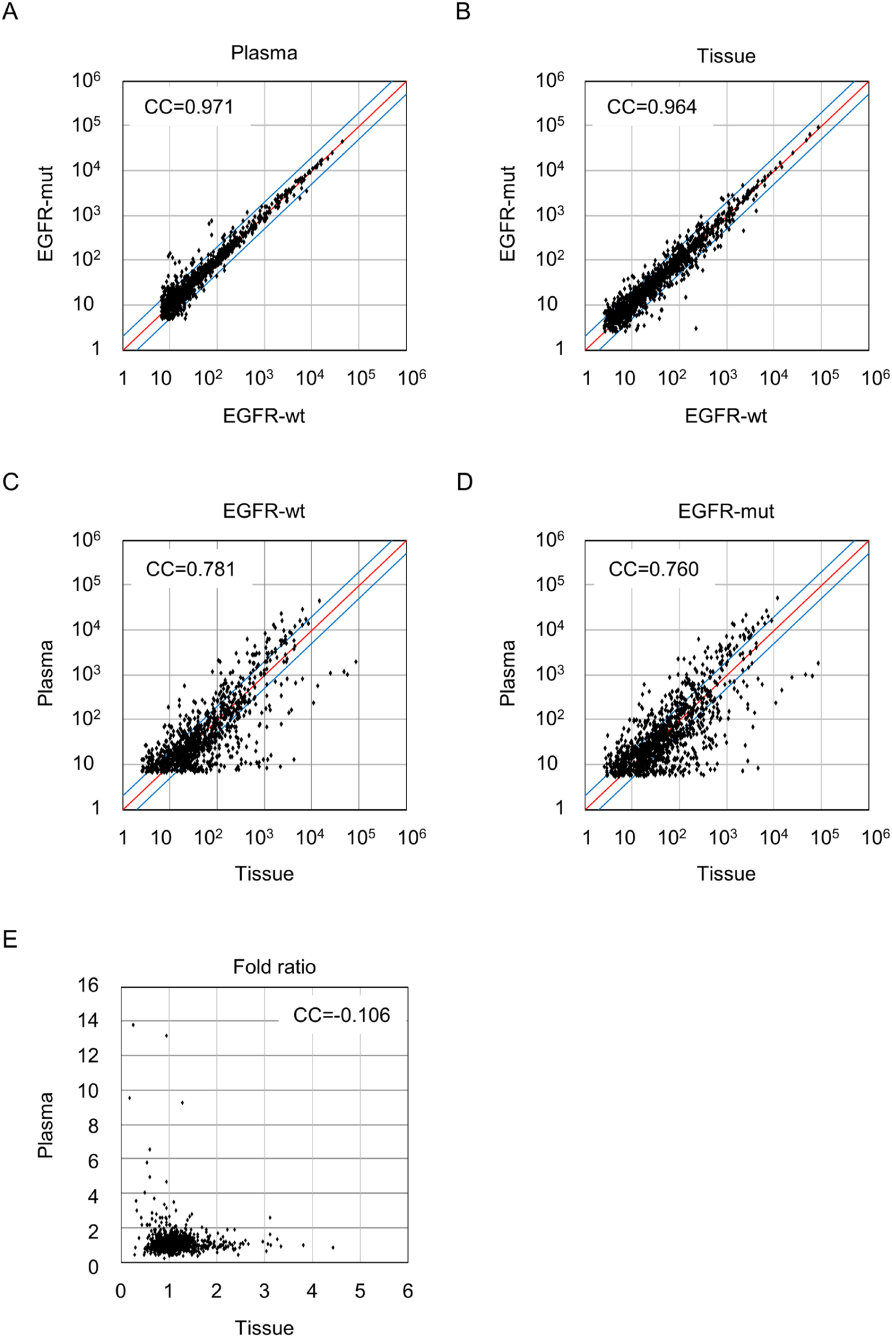
Pairwise correlation comparison of miRNA expression profiles between EGFR-wt and EGFR-mut in plasma and tumor tissues Intensity scatter plots of plasma **(A)** and tissue **(B)** derived miRNA expression for EGFR-wt versus EGFR-mut. Intensity scatter plots of miRNA expression for plasma miRNA versus tissue miRNAs in EGFR-wt **(C)** and in EGFR-mut **(D)**. **(E)** indicates scatter plots of miRNA fold ratio of EGFR-mut/EGFR-wt between plasma and tissue. Correlation coefficients (CC) for each comparison are shown.

Clinicopathological characteristics are shown in Table 1. To choose miRNAs which shows statistically significant different expression according to EGFR mutational status in smoker males with early stage of lung adenocarcinoma, we attempted to obtain comparative miRNA profiles using paired plasma and tumor tissues from same patients. Microarray was performed using paired plasma and tissue samples, each was pooled from 10 EGFR-wt and 10 EGFR-mut patients respectively, of both were diagnosed as stage I by pTNM staging. The correlation coefficient of plasma miRNA profiles was 0.971, indicating that EGFR-wt patients and EGFR-mut patients share a similar miRNA expression repertoire (Figure 1A). The similar trend was also observed for tissue miRNAs (Figure 1B). Further, in both EGFR-wt and EGFR-mut subjects, although strong correlation was found between plasma and tissue miRNA intensity per se (Figure 1C and 1D), the ratios of EGFR-mut/EGFR-wt miRNA between plasma and tissues showed no correlation in our samples (Figure 1E), indicating that expression ratio between plasma miRNA and tissue miRNA is extremely differed even though contents of both miRNA profile are similar. We sorted these miRNAs with respect to their hybridization intensity ratio and selected 15 miRNAs from plasma miRNA profiles and 2 miRNAs from tissue miRNA profiles among miRNAs with a two-fold or higher intensity ratio (Supplementary Table 1 and 2). As shown in Table 2, while 5 miRNAs (miR-192-5p, miR-194-5p, miR-346, miR-4704-3p, miR-6765-3p) were expressed higher in pooled EGFR-wt patients, 12 miRNAs (miR-16-5p, miR-23a-3p, miR-92b-3b, miR-103a-3p, miR-122-5p, miR-223-3p, miR-451a, miR-619-5p, miR-1246, miR-1290, miR-4732-5p, miR-6778-5p) were expressed higher in pooled EGFR-mut patients.

**Table 1:** Patients characteristics and EGFR mutation status

Variables	No.	EGFR status wt / mut (%)	*P*-value
Age (y)			
≤70	44	28 / 16 (63.6% / 36.4%)	0.59
>70	39	27 / 12 (69.2% / 30.8%)	
Smoking history			
Never smoker	15	4 / 11 (26.7% / 73.3%)	<0.0003
Smokers	68	51 / 17 (75.0% / 25.0%)	
≤30 pack-years (15)		5 / 10 (33.3% / 66.7%)	
>30 pack-years (48)		41 / 7 (85.4% / 14.6%)	
unknown (5)		5 / 0 (100% / 0%)	
Disease stage			
Stage I	52 (45^*^)	35 / 17 (67.3% / 32.7%)	0.96
Stage II	20 (13^*^)	13 / 7 (65.0% / 35.0%)	
Stage III	11 (10^*^)	7/ 4 (63.6% / 36.4%)	

^*^ indicates the number of smoker patients.

**Table 2:** Microarray analysis revealed 17 differentially expressed miRNAs over 2-fold in different EGFR status

Signal intensity (Global normalization, ratio)				
miRNA	EGFR-wt	EGFR-mut	origin	wt/mut
miR-16-5p	10.20	140.30	plasma	0.07
miR-23a-3p	12.90	83.90	plasma	0.15
miR-92b-3p	41.60	166.70	plasma	0.25
miR-103a-3p	ND	47.30	plasma	-
miR-122-5p	11.60	110.20	plasma	0.11
miR-192-5p	140.20	14.40	tissue	9.74
miR-194-5p	222.90	2.90	tissue	76.86
miR-223-3p	ND	80.70	plasma	-
miR-346	21.40	5.10	plasma	4.20
miR-451a	78.50	749.70	plasma	0.10
miR-619-5p	142.60	370.90	plasma	0.38
miR-1246	67.50	623.40	plasma	0.11
miR-1290	9.50	124.10	plasma	0.08
miR-4704-3p	48.40	ND	plasma	-
miR-4732-5p	30.80	107.50	plasma	0.29
miR-6765-3p	140.50	57.20	plasma	2.46
miR-6778-5p	75.50	350.40	plasma	0.22

### Confirmation of candidate miRNAs by real-time qRT-PCR

**Figure 2:**
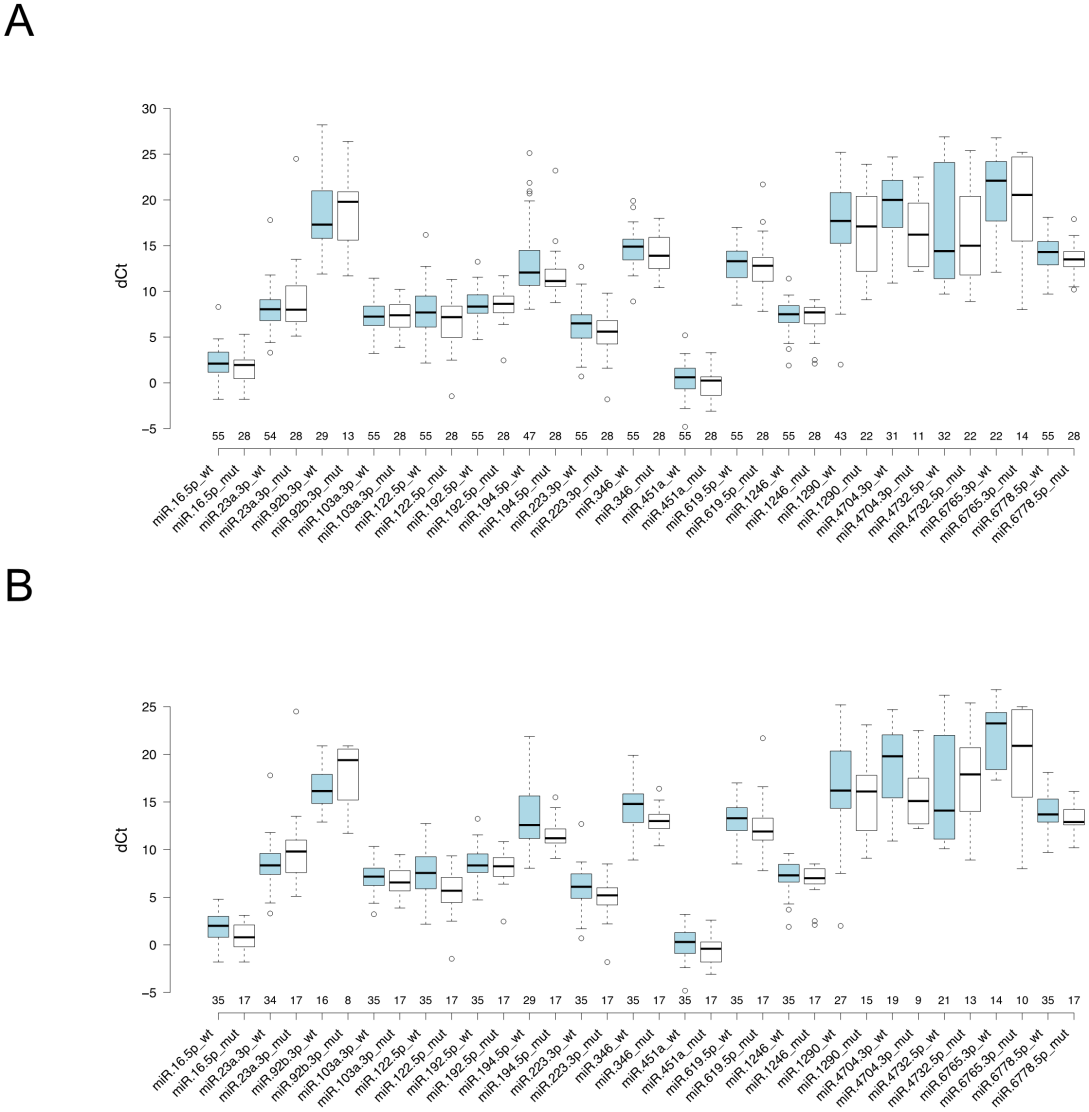
Boxplot of qRT-PCR results of selected 17 miRNAs Expression patterns for each EGFR-wt and EGFR-mut patients are shown. **(A)** 83 NSCLC all stage patients and **(B)** 52 NSCLC stage I patients. Vertical axis shows the dCt (Ct _test_ - Ct _ath-miR-159a_) value. Sample numbers whose expression were detected within 40 cycles by qRT-PCR are indicated. The circles represent the outliers. *P*-values are indicated in Table 3.

To confirm the expression patterns of 17 candidate miRNAs, real-time qRT-PCR (hereafter referred to as qRT-PCR) for each miRNA was performed with 83 plasma samples consisting of 55 EGFR-wt and 28 EGFR-mut patients. Here, we included the patients with non-smoking history to investigate the association of miRNA expressions with smoking history. The result confirmed that elevated expression of miR-192-5p (p=0.807) and reduced expressions of miR-16-5p (p=0.19), miR-23a-3p (p=0.373), miR-122-5p (p=0.093), miR-223-3p (p=0.168), miR-451a (p=0.174), miR-619-5p (p=0.44), miR-1290 (p=0.487), and miR-6778-5p (p=0.101) in EGFR-wt patients compared with EGFR-mut patients were consistent with the results obtained from microarray analysis (Table 3, Figure 2A). On the other hand, the expression patterns of other miRNAs, elevated expressions of miR-92b-3p (p=0.687), miR-103a-3p (p=0.928), miR-1246 (p=0.663), and miR-4732-5p (p=0.594) and reduced expressions of miR-194-5p (p=0.066), miR-346 (p=0.302), miR-4704-3p (p=0.051), and miR-6765-3p (p=0.532) in EGFR-wt patients compared with EGFR-mut patients, was not consistent with those shown by microarray analysis. The result indicated that about half of 17 miRNAs showed similar expression tendency in both assays. The lack of consistency in two analyses was considered due to the difference in detection platforms between microarray (hybridization) and qRT-PCR. The result of qRT-PCR analysis showed that there was no statistically significant difference for any of the 17 miRNAs between EGFR-wt patients and EGFR-mut patients when looking at all stages of disease.

**Table 3:** qRT-PCR quantification of 17 miRNAs in all 83 patients with stage stratification

All patients	All stage (83)			Stage I (52)			Stage I+II (72)			Stage II+III (31)		
miRNA	wt (55)	mut (28)	*P*-value	wt (35)	mut (17)	*P*-value	wt (48)	mut (24)	*P*-value	wt (20)	mut (11)	*P*-value
miR-16-5p	2.1	2.0	0.190	2.0	0.8	**0.038**	2.2	1.7	0.061	2.8	2.5	0.887
miR-23a-3p	8.0	8.0	0.373	8.4	9.8	0.207	8.1	8.6	0.268	7.4	7.4	0.951
miR-92b-3p	17.3	19.8	0.687	16.2	19.4	0.320	17.1	19.8	0.494	21.3	21.9	0.924
miR-103a-3p	7.2	7.4	0.928	7.2	6.5	0.462	7.4	7.2	0.495	7.7	8.4	0.611
miR-122-5p	7.7	7.2	0.093	7.6	5.7	**0.003**	7.6	6.7	**0.041**	8.1	8.5	0.338
miR-192-5p	8.3	8.6	0.807	8.3	8.2	0.486	8.3	8.6	0.771	8.5	8.9	0.381
miR-194-5p	12.1	11.1	0.066	12.6	11.2	**0.037**	12.3	11.2	0.092	11.8	11.0	0.707
miR-223-3p	6.5	5.6	0.168	6.1	5.2	0.110	6.5	5.6	0.066	6.8	6.3	0.887
miR-346	14.9	13.9	0.302	14.8	13.0	**0.017**	14.9	13.6	0.141	15.1	16.0	0.095
miR-451a	0.6	0.2	0.174	0.3	−0.4	0.055	0.6	0.04	0.070	1.1	0.5	0.951
miR-619-5p	13.3	12.8	0.440	13.3	11.9	0.129	13.3	13.0	0.487	12.5	13.1	0.611
miR-1246	7.5	7.7	0.663	7.3	7.0	0.333	7.5	7.5	0.473	7.6	7.9	0.476
miR-1290	17.7	17.1	0.487	16.2	16.1	0.362	17.7	16.9	0.383	19.6	21.8	0.535
miR-4704-3p	20.0	16.2	0.051	19.8	15.1	0.085	20.4	15.7	0.053	20.4	21.1	0.549
miR-4732-5p	14.4	15.0	0.594	14.1	17.9	0.675	14.4	15.2	0.772	23.4	12.0	0.131
miR-6765-3p	22.1	20.5	0.532	23.3	20.9	0.312	22.1	20.5	0.307	21.3	20.4	1.000
miR-6778-5p	14.3	13.5	0.101	13.7	12.9	0.102	14.3	13.3	0.074	14.8	13.7	0.427

The expression levels of miRNAs were calculated by using the delta Ct method (dCt = Ct _test_ - Ct _ath-miR-159a_). Each median value is shown.

The *P*-values were determined by Mann-Whitney U test. Bold: P<0.05.

Since microarray analysis was done using only stage I lung adenocarcinoma specimens, we reanalyzed these 17 miRNAs qRT-PCR data of 83 lung adenocarcinoma samples with stratification by stage at diagnosis. The result revealed that the expression levels of four miRNAs, miR-16-5p (p=0.038), miR-122-5p (p=0.003), miR-194-5p (p=0.037) and miR-346 (p=0.017) were statistically significant higher in mutated EGFR patients compared with EGFR-wt patients in disease stage I (Table 3, Figure 2B).

### Stratification of miRNAs by smoking status and pTNM stage classification

**Figure 3:**
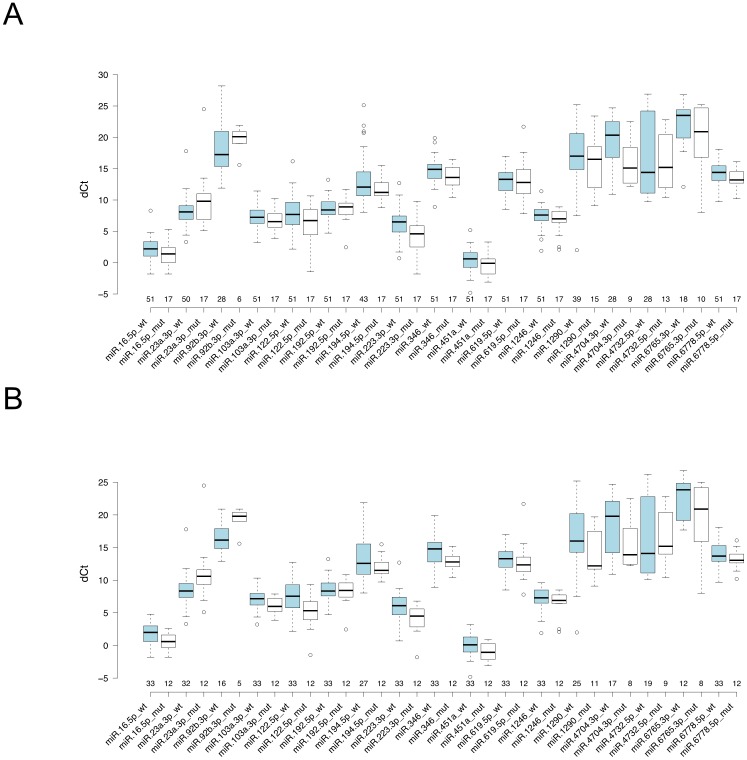
Boxplot of qRT-PCR results of selected 17 miRNAs for smoker patients Expression patterns for each smoker patient are shown. **(A)** 68 NSCLC all stage patients and **(B)** 45 NSCLC stage-I patients. Vertical axis shows the dCt (Ct _test_ - Ct _ath-miR-159a_) value. Number of samples detected within 40 cycles by qRT-PCR are shown. The circles represent the outliers. *P*-values are indicated in Table 4.

To determine whether selected 17 miRNAs show a positive correlation with smoking and disease progression, we classified the results of qRT-PCR by smoking status and disease stages (I-III). After exclusion of 15 non-smoker patients, a total of 68 smoker lung adenocarcinoma patients consisted of 45 cases of stage I, 13 cases of stage II and 10 cases of stage III (Table 1) were further analyzed. Initially, when the correlation of 17 miRNA expressions with EGFR status was examined in smokers of all stages, significant differences were found in two miRNAs, miR-122-5p (p=0.048) and miR-223-3p (p=0.012), which showed higher expression in EGFR mutated patients compared with EGFR-wt patients (Table 4, Figure 3A). Next, stratification of miRNA expressions by disease stage revealed that in both stage I and stage I+II disease, six miRNAs, miR-16-5p (p=0.023), miR-103a-3p (p=0.042), miR-122-5p (p=0.006), miR-223-3p (p=0.020), miR-346 (p=0.017) and miR-451a (p=0.038) showed higher expression in EGFR-mut patients compared with EGFR-wt patients, whereas expression of miR-23a-3p (p=0.009) was higher in EGFR-wt patients (P values of either stage I or stage I/II are exhibited) (Table 4, Figure 3B). Of note, statistical significance of miR-23a-3p, miR-103a-3p, miR-223-3p and miR-451a were only seen when smoker patients were subjected to analysis, indicating that expressions of these miRNAs are associated with smoking. On the other hand, significance of miR-194-5p expression in EGFR-mut patients including both smoker and non-smoker patients were disappeared in smoker patients, indicating that this miRNA is not associated with smoking status. Among smokers, no significant differences were seen between current- and former-smokers in all 17 miRNAs expression level. It was the same for stratified early stage groups, with the exception of miR-16-5p and miR-451a of smoker stage I group (p=0.03 and 0.0499, respectively; Supplementary Table 3). Interestingly, the expression levels of miR-16-5p, miR-122-5p and miR-346 showed reverse trend between EGFR-wt patients and EGFR-mut patients as disease stage advanced, however statistical significance was not observed due to small size of EGFR-mut patients with stage II and stage III disease.

**Table 4:** qRT-PCR quantification of 17 miRNAs in 68 smoker patients with stage stratification

Smokers	All stage (68)			Stage I (45)			Stage I+II (58)			Stage II+III (23)		
miRNA	wt (51)	mut (17)	*P*-value	wt (33)	mut (12)	*P*-value	wt (44)	mut (14)	*P*-value	wt (18)	mut (5)	*P*-value
miR-16-5p	2.2	1.4	0.104	2.0	0.6	**0.023**	2.3	1.0	**0.020**	3.0	3.0	0.587
miR-23a-3p	8.1	9.8	0.060	8.4	10.6	**0.009**	8.1	10.1	**0.022**	7.7	6.5	0.538
miR-92b-3p	17.3	20.1	0.318	16.2	19.8	0.062	16.9	19.8	0.215	21.4	21.9	0.923
miR-103a-3p	7.2	6.5	0.158	7.2	6.0	0.055	7.4	6.4	**0.042**	8.0	8.6	0.745
miR-122-5p	7.7	6.7	**0.048**	7.5	5.3	**0.006**	7.6	5.9	**0.011**	8.4	10.3	0.403
miR-192-5p	8.4	8.9	0.736	8.3	8.4	0.752	8.4	8.7	0.713	8.7	9.3	0.290
miR-194-5p	12.1	11.2	0.215	12.6	11.5	0.284	12.3	11.5	0.288	11.8	9.9	0.398
miR-223-3p	6.5	4.6	**0.012**	6.1	4.5	**0.020**	6.5	4.5	**0.003**	6.9	5.9	0.491
miR-346	14.9	13.6	0.063	14.8	12.8	**0.017**	14.9	13.0	**0.019**	15.1	15.8	0.325
miR-451a	0.6	−0.1	0.104	0.1	−1.1	**0.038**	0.8	−0.3	**0.027**	1.1	0.6	0.857
miR-619-5p	13.3	12.8	0.663	13.3	12.4	0.341	13.3	13.0	0.836	12.8	14.9	0.857
miR-1246	7.6	7.0	0.199	7.3	6.9	0.303	7.7	6.9	0.172	7.7	7.6	0.638
miR-1290	17.0	16.5	0.212	16.0	12.2	0.292	17.5	15.6	0.119	19.6	18.7	0.878
miR-4704-3p	20.4	15.1	0.057	19.8	13.9	0.157	20.7	13.9	0.060	20.7	20.9	0.833
miR-4732-5p	14.4	15.2	0.628	14.1	15.2	1.000	14.4	15.2	0.740	24.0	14.1	0.414
miR-6765-3p	23.5	20.9	0.332	23.9	20.9	0.208	23.5	20.6	0.136	22.3	22.8	1.000
miR-6778-5p	13.2	14.4	0.089	13.0	13.7	0.207	13.0	14.4	0.059	13.8	14.9	0.325

The expression levels of miRNAs were calculated by using the delta Ct method (dCt = Ct _test_ - Ct _ath-miR-159a_). Each median value is shown.

The *P*-values were determined by Mann-Whitney U test. Bold: P<0.05.

## DISCUSSION

Since the molecular target therapy against NSCLC patients harboring somatic mutations in EGFR genes has been evolved, clinical sequencing for mutations in EGFR gene is therefore an important step in the treatment-decision pathway [26]. Expression profiling of miRNAs associated with EGFR mutational status in tumor tissues and bloodstream have been extensively investigated to translate specific miRNAs as prediction biomarker [18, 25, 27, 28]. However, there are still limited studies regarding to circulating miRNA expression signatures which enable to distinguish mutation status of EGFR gene in lung adenocarcinoma male patients with smoking history. Therefore, identification and validation of such miRNA signatures as a diagnostic tool is important to decide whether those patients get TKI treatment prior to surgical operation to improve the outcome.

In this study, we used miRNA microarray analysis in initial screening of miRNAs which differentially expressed in smoker male patients with stage I lung adenocarcinoma harboring either wild type or mutated EGFR genes. In initial screening, we found that total 84 circulating miRNAs were differentially expressed with a two-fold or higher intensity ratio in either EGFR-wt patients or EGFR-mut patients. Because of limited availabilities of Exiqon miRNA qPCR primers as well as published literatures, we selected 17 miRNAs and subsequently confirmed their specificity in increased number of plasma samples consisting of disease stage I to III by qRT-PCR analysis. The results revealed that no miRNA was confirmed a significant difference between the EGFR status with the usual 5% significance level and the expression levels of miRNAs were rather reversed between EGFR-wt patients and EGFR-mut patients in many cases when compared miRNA microarray with qRT-PCR assay (Table 2, Table 3). One possible reason for this controversial result is considered that patient(s) representing the outliers which was far from the median value of total 83 patients was included in pooled samples used for initial screening by microRNA microarray. In fact, the result of one patient (72 years old, smoker, EGFR-mut L858R) showed extremely higher expression of almost all miRNAs than other patients in qRT-PCR. Another possible reason is that the expression level of some miRNAs is closely related to cancer progression [29, 30]. For example, Tanaka Y, et al. have reported that in esophageal squamous cell carcinoma, expression of circulating exosomal miR-21 was correlated with advanced tumor classification, positive lymph node status, and the presence of metastasis with inflammation and clinical stage without inflammation [31]. Therefore, the large difference in miRNA expressions among diagnostic stages is also as one of factors that caused inconsistency in the results from miRNA microarray assay (only stage I) and qRT-PCR assay (including stage I, II and III). Stratified analysis of stage I patients revealed significant higher expression of 4 out of 17 miRNAs in EGFR-mut group (miR-16-5p, miR-122-5p, miR-194-5p and miR-346). Further stratified analysis of smoker stage I patients successfully identified additional 3 miRNAs, miR-23a-3p, miR-223-3p and miR-451a as smoking responsive miRNA in early stage lung adenocarcinoma and upregulation of miR-23a-3p was strongly higher in EGFR-wt genotype while upregulation of miR-223-3p and miR-451a were significantly higher in EGFR-mut genotype. This is the first study suggesting the diagnostic value of these miRNA as potential biomarkers whose alteration would be able to distinguish EGFR gene mutation status of male smoker patients with early stage lung adenocarcinoma.

In agreement with our observation, several studies have recently revealed upregulation of miR-23a, miR-194 and miR-223 in plasma and oral mucosa from smokers with lung adenocarcinoma and plasma miR-223-3p has been shown significant association with a higher risk for disease progression [12, 32, 33]. Further, miR-23a and miR-192 are known to express higher in male patients than female patients, indicating their gender specificity [33]. On the other hands, meta-analysis of miRNA expressions in lung cancer tissues has found downregulation of miR-451a as the disease progresses, which acts as tumor suppressor miRNA in gastric cancer and melanoma, however, its expression in body fluids was not examined and also not associated with smoking [18, 34–37]. Importantly, none of these studies have succeeded to show the association of expression of these miRNAs with EGFR mutational status. Therefore, our finding of smoking and EGFR mutation associated miRNA signature (miR-23a-3p, miR-223-3p and miR-451a) shed light on their biological importance in EGFR signaling pathway in lung adenocarcinoma development and progression affected by smoking habit.

It has been reported that miR-16-5p, miR-122-5p, miR-194 and miR-451a inhibit lung adenocarcinoma development by suppressing proliferation, invasion, and metastasis through targeting different cancer associated genes [38–42]. The miR-346 has been shown to be involved in proliferation, invasion, and drug resistance of lung adenocarcinoma by positively regulating the XPC/ERK/Snail/E-cadherin pathway [43, 44]. let-7 miRNAs which generally play a tumor-suppressive role as targeting oncogenes such as RAS and HMGA2 is known to be selectively secreted into extracellular environment via exosomes to maintain tumorigenic and metastatic propensities of gastric cancer cells [45]. Consistent with our results of increased amounts of those miRNAs in plasma from lung adenocarcinoma patients, several studies have similarly detected upregulation of tumor suppressor miRNAs in body fluid in different types of cancer such as gastric cancer and breast cancer [46, 47]. Thus, observation of elevated expression of such miRNAs in plasma in this study also suggests that their secretion from tumor tissue would probably promote tumorigenesis of lung adenocarcinoma. In addition, it has more recently been found that tumor suppressor gene induced senescent cells modulate immune response which promotes establishment of the inflammatory microenvironment which contributes to metastasis [48]. While miRNA-194, a typical p53 responsive miRNA has been shown to trigger the replicative senescence of MEF cells by potentially inhibiting the DNMT3A expression [49], miR-122-5p has been identified as senescence-associated miRNA in normal human lung fibroblasts [50]. Therefore, tumor suppressor miRNAs discharged from lung adenocarcinoma also may play a major role in induction of senescence of cells around proximal and distant tissues to promote the metastasis of lung adenocarcinoma to brain, bone and liver.

We are aware of the limitation of our study. First, some data was censored; sample size of the patients with advanced stages was relatively small. In addition, we did not investigate the mutations of KRAS and ALK genes in EGFR-wt patients, which play a major role in the progression of lung adenocarcinoma and are mutually exclusive from EGFR gene mutations [51]. Therefore, a further study will be needed to confirm whether or not miRNA signatures identified in this study is associated with higher risk for disease progression and whether or not upregulation of miR-23a-3p is associated with other major genetic alterations.

In conclusion, in this explorative study, we identified circulating miRNA signature with significantly different expression associated with EGFR gene mutational status in Japanese smoker males with lung adenocarcinoma. Further investigation is essential to develop new miRNA signatures as discriminable non-invasive prediction biomarker of mutation status of EGFR gene in smoker male with lung adenocarcinoma and other tumors types and also to evaluate whether the findings is applicable to smoker male patients of other races.

## MATERIALS AND METHODS

### Clinical samples

All patients in this study were recruited from Okayama University Hospital between 2010 and 2015. The patients were newly diagnosed and histologically confirmed primary lung adenocarcinoma. The patient characteristics are shown in Table 1. The male lung adenocarcinoma specimens consisted of 55 EGFR-wt patients and 28 EGFR-mut patients, and their median age was 70. 52 patients had stage I disease, 20 stage II, and 11 stage III. 68 cases were from ever smoker with a median smoke exposure of 45 pack-years (29 cases were current smokers and 39 cases were former smokers) and 15 cases were never smokers. Five ever smokers had no pack-years data. 83 plasma samples consisting of 55 EGFR-wt and 28 EGFR-mut and surgically resected specimens of 20 lung adenocarcinomas consisting of 10 EGFR-wt and 10 EGFR-mut were obtained from male lung adenocarcinoma patients. Plasmas from patients with a previous medical history of cancer, radiotherapy or chemotherapy before surgery were pre-excluded. All patients were given written informed consent. The study was approved by the Bioethics Committee of Okayama University Medical School.

### MicroRNA extraction

MiRNA was extracted from 200 μl of plasma samples using the *mir*Vana PARIS Protein and RNA Isolation System (Thermo Fisher Scientific) according to the manufacturer’s instructions. Since no miRNA has been established as a house-keeping gene in the plasma, we added 4 fmol of synthetic Arabidopsis thaliana miRNA ath-miR-159a (plasma spiked-in control) to each plasma sample as an external control to monitor the quality of RNA extraction and analysis. From lung adenocarcinoma tissue samples, total RNA containing small RNA was extracted using TRIsol® reagent (Thermo Fisher Scientific) according to the manufacturer’s protocol, and the concentrations were determined using Nano drop (Thermo Fisher Scientific). The total RNA extracts were stored at −80°C until required.

### MicroRNA profiling by microarray

The blood plasmas were pooled from 10 EGFR-wt patients and 10 EGFR-mut patients respectively for microarray analysis. Total RNA containing small RNA was also pooled equally for each 10 lung adenocarcinoma tissues as well as plasmas. MiRNA profiling was examined using a Toray 3D-Gene® miRNA oligo chip Human miRNA version 21 (Toray) on which 2,565 probes were mounted. The expression level of each miRNA was normalized using the median signal strength for the entire gene in each chip. Raw data was deposited to Gene Expression Omnibus (GEO Accession No. GSE102222 and GSE102223).

### Detection of plasma miRNA by qRT-PCR

For miRNA quantitative RT-PCR assay, we used the Locked Nucleic Acid (LNA)-based miRNA qPCR platform from Exiqon: hsa-miR-16-5p, hsa-miR-23a-3p, hsa-miR-92b-3p, hsa-miR-103a-3p, hsa-miR-122-5p, hsa-miR-192-5p, hsa-miR-223-3p, hsa-miR-346, hsa-miR-451a, hsa-miR-619-5p, hsa-miR-1246, hsa-miR-1290, hsa-miR-4704-3p, hsa-miR-4732-5p, hsa-miR-6765-3p and hsa-miR-6778-5p. Total RNA was used for cDNA synthesis using MiRCURY LNA Universal cDNA Synthesis Kit II (Exiqon). The cDNA was diluted and qRT-PCR was carried out using specific, pre-defined microRNA primer pairs and the ExiLENT SYBR Green Master Mix (Exiqon) using an ABI7300 instrument (Applied Biosystems). Two miRNAs (hsa-miR-194-5p and ath-miR-159a) were quantified using TaqMan MicroRNA Assay Kit. All steps were performed following the manufacturer’s protocol. To identify the differentially expressed miRNAs, the expression levels were calculated with Delta Ct method (Delta Ct=Ct _test_-Ct_ath-miR-159a_).

### Statistical analysis

The categorical variables of EGFR status were compared using the chi square or Fisher’s exact test, as appropriate. Statistical analysis of miRNA expression was performed using Mann-Whitney U test to determine any significant difference between two groups. *P*-values < 0.05 were considered statistical significant. SPSS (IBM) was used for statistical analysis.

## SUPPLEMENTARY MATERIALS FIGURES AND TABLES









## Abbreviations

EGFR: epidermal growth factor receptor
NSCLC: non-small cell lung cancer
miRNA: microRNA
qRT-PCR: quantitative real-time polymerase chain reaction
wt: wild type
mut: mutated
