# Supplementary material for: Unique circulating microRNAs in relation to EGFR mutation status in Japanese smoker male with lung adenocarcinoma
Source: Oncotarget. 2017 Sep 30;8(70):114685–97. doi: 10.18632/oncotarget.21425 (PMC5777724; doi:10.18632/oncotarget.21425)
Supplement: Supplementary file 1 [file oncotarget-08-114685-s001.pdf]

# Unique circulating microRNAs in relation to EGFR mutation status in Japanese smoker male with lung adenocarcinoma

## SUPPLEMENTARY MATERIALS

**Supplementary Table 1: miRNAs expressed in EGFR-wt more than 2-fold as compared with EGFR-mut by microarray**

See Supplementary File 1

**Supplementary Table 2: miRNAs expressed in EGFR-mut more than 2-fold as compared with EGFR-wt by microarray**

See Supplementary File 2

**Supplementary Table 3: Comparison of 17 miRNAs expression between current- and former-smoker**

See Supplementary File 3
