# Supplementary material for: Unique circulating microRNAs in relation to EGFR mutation status in Japanese smoker male with lung adenocarcinoma
Source: Oncotarget. 2017 Sep 30;8(70):114685–97. doi: 10.18632/oncotarget.21425 (PMC5777724; doi:10.18632/oncotarget.21425)
Supplement: Supplementary file 2 [file oncotarget-08-114685-s002.docx]

Supplementary Table 1 miRNAs expressed in EGFR-wt more than 2-fold as compared with EGFR-mut by microarray.

| **Plasma** |  | **Global normalization（ratio）** | |  |
| --- | --- | --- | --- | --- |
| **Name** | **ID** | **wt** | **mut** | **Log2Ratio** |
| hsa-miR-579-5p | MIMAT0026616 | 38.7 | - | - |
| hsa-miR-4704-3p | MIMAT0019804 | 48.4 | - | - |
| hsa-miR-346 | MIMAT0000773 | 21.4 | 5.1 | 2.08 |
| hsa-miR-1295b-3p | MIMAT0022294 | 21.9 | 6.1 | 1.85 |
| hsa-miR-6783-5p | MIMAT0027466 | 18.1 | 6.0 | 1.60 |
| hsa-miR-7110-3p | MIMAT0028118 | 25.8 | 9.6 | 1.42 |
| hsa-miR-6861-3p | MIMAT0027624 | 19.6 | 7.4 | 1.41 |
| hsa-miR-5196-3p | MIMAT0021129 | 15.1 | 6.0 | 1.33 |
| hsa-miR-6731-3p | MIMAT0027364 | 20.6 | 8.3 | 1.32 |
| hsa-miR-6749-3p | MIMAT0027399 | 36.1 | 14.6 | 1.31 |
| hsa-miR-4316 | MIMAT0016867 | 53.4 | 21.6 | 1.30 |
| hsa-miR-4785 | MIMAT0019949 | 14.8 | 6.0 | 1.30 |
| hsa-miR-6765-3p | MIMAT0027431 | 140.5 | 57.2 | 1.30 |
| hsa-miR-1236-5p | MIMAT0022945 | 14.5 | 6.0 | 1.27 |
| hsa-miR-6087 | MIMAT0023712 | 5792.5 | 2457.6 | 1.24 |
| hsa-miR-4642 | MIMAT0019702 | 20.3 | 8.7 | 1.22 |
| hsa-miR-4708-5p | MIMAT0019809 | 11.6 | 5.0 | 1.20 |
| hsa-miR-525-5p | MIMAT0002838 | 17.2 | 7.5 | 1.20 |
| hsa-miR-202-3p | MIMAT0002811 | 12.2 | 5.4 | 1.17 |
| hsa-miR-4532 | MIMAT0019071 | 7979.8 | 3569.3 | 1.16 |
| hsa-miR-1288-5p | MIMAT0026743 | 12.6 | 5.7 | 1.16 |
| hsa-miR-1185-2-3p | MIMAT0022713 | 98.6 | 45.3 | 1.12 |
| hsa-miR-6895-3p | MIMAT0027691 | 16.6 | 7.7 | 1.12 |
| hsa-miR-449b-3p | MIMAT0009203 | 20.6 | 9.6 | 1.11 |
| hsa-miR-4285 | MIMAT0016913 | 15.0 | 7.1 | 1.08 |
| hsa-miR-5008-3p | MIMAT0021040 | 13.5 | 6.4 | 1.07 |
| hsa-miR-210-3p | MIMAT0000267 | 10.2 | 4.9 | 1.07 |
| hsa-miR-1185-1-3p | MIMAT0022838 | 146.0 | 70.7 | 1.05 |
| hsa-miR-550a-3-5p | MIMAT0020925 | 33.9 | 16.5 | 1.04 |
| hsa-miR-7111-3p | MIMAT0028120 | 17.5 | 8.5 | 1.04 |
| hsa-miR-527,  hsa-miR-518a-5p | MIMAT0002862,  MIMAT0005457 | 20.3 | 10.0 | 1.03 |
| hsa-miR-3943 | MIMAT0018359 | 9.9 | 4.9 | 1.01 |
| hsa-miR-6802-3p | MIMAT0027505 | 15.6 | 7.8 | 1.01 |
| **Tissue** | | | | |
| hsa-miR-192-5p | MIMAT0000222 | 140.2 | 14.4 | 3.28 |
| hsa-miR-194-5p | MIMAT0000460 | 222.9 | 2.9 | 6.26 |
| hsa-miR-215-5p | MIMAT0000272 | 47.0 | - | - |
| hsa-miR-373-5p | MIMAT0000725 | 38.9 | - | - |
| hsa-miR-5584-3p | MIMAT0022284 | 34.9 | - | - |
| hsa-miR-31-5p | MIMAT0000089 | 42.9 | 7.8 | 2.46 |
| hsa-miR-203a-3p | MIMAT0000264 | 26.2 | 5.6 | 2.23 |
| hsa-miR-223-3p | MIMAT0000280 | 314.8 | 57.0 | 2.47 |
| hsa-miR-126-5p | MIMAT0000444 | 41.4 | 7.8 | 2.41 |
| hsa-miR-374a-5p | MIMAT0000727 | 13.1 | 2.9 | 2.18 |
| hsa-miR-338-3p | MIMAT0000763 | 37.7 | 5.1 | 2.89 |
| hsa-miR-451a | MIMAT0001631 | 2314.5 | 434.5 | 2.41 |
| hsa-miR-660-5p | MIMAT0003338 | 18.1 | 3.5 | 2.37 |
| hsa-miR-362-3p | MIMAT0004683 | 12.4 | 2.7 | 2.20 |
| hsa-let-7a-5p | MIMAT0000062 | 1111.8 | 466.1 | 1.25 |
| hsa-let-7d-5p | MIMAT0000065 | 779.4 | 363.3 | 1.10 |
| hsa-let-7e-5p | MIMAT0000066 | 334.7 | 121.3 | 1.46 |
| hsa-let-7f-5p | MIMAT0000067 | 662.4 | 204.8 | 1.69 |
| hsa-miR-15a-5p | MIMAT0000068 | 157.8 | 47.7 | 1.73 |
| hsa-miR-16-5p | MIMAT0000069 | 761.4 | 197.4 | 1.95 |
| hsa-miR-17-5p | MIMAT0000070 | 193.8 | 96.6 | 1.00 |
| hsa-miR-20a-5p | MIMAT0000075 | 145.5 | 54.2 | 1.42 |
| hsa-miR-26b-5p | MIMAT0000083 | 240.9 | 69.9 | 1.79 |
| hsa-miR-27a-3p | MIMAT0000084 | 609.5 | 288.2 | 1.08 |
| hsa-miR-98-5p | MIMAT0000096 | 37.3 | 13.7 | 1.44 |
| hsa-miR-99a-5p | MIMAT0000097 | 216.4 | 98.9 | 1.13 |
| hsa-miR-29b-3p | MIMAT0000100 | 790.7 | 326.7 | 1.28 |
| hsa-miR-106a-5p | MIMAT0000103 | 227.1 | 92.3 | 1.30 |
| hsa-miR-199a-3p,  hsa-miR-199b-3p | MIMAT0000232,  MIMAT0004563 | 324.8 | 162.0 | 1.00 |
| hsa-miR-30c-5p | MIMAT0000244 | 372.0 | 161.1 | 1.21 |
| hsa-miR-183-5p | MIMAT0000261 | 7.6 | 3.8 | 1.00 |
| hsa-miR-199b-5p | MIMAT0000263 | 65.7 | 31.8 | 1.05 |
| hsa-miR-212-3p | MIMAT0000269 | 20.4 | 9.1 | 1.16 |
| hsa-miR-218-5p | MIMAT0000275 | 12.3 | 5.6 | 1.14 |
| hsa-let-7g-5p | MIMAT0000414 | 226.5 | 77.2 | 1.55 |
| hsa-let-7i-5p | MIMAT0000415 | 227.5 | 104.6 | 1.12 |
| hsa-miR-15b-5p | MIMAT0000417 | 110.9 | 35.7 | 1.64 |
| hsa-miR-142-5p | MIMAT0000433 | 115.3 | 45.9 | 1.33 |
| hsa-miR-142-3p | MIMAT0000434 | 88.4 | 30.3 | 1.54 |
| hsa-miR-144-3p | MIMAT0000436 | 48.3 | 14.6 | 1.73 |
| hsa-miR-126-3p | MIMAT0000445 | 552.1 | 174.5 | 1.66 |
| hsa-miR-146a-5p | MIMAT0000449 | 127.1 | 46.6 | 1.45 |
| hsa-miR-150-5p | MIMAT0000451 | 192.2 | 72.2 | 1.41 |
| hsa-miR-195-5p | MIMAT0000461 | 184.1 | 83.2 | 1.15 |
| hsa-miR-206 | MIMAT0000462 | 8.3 | 4.1 | 1.02 |
| hsa-miR-155-5p | MIMAT0000646 | 58.3 | 26.8 | 1.12 |
| hsa-miR-29c-3p | MIMAT0000681 | 617.7 | 299.7 | 1.04 |
| hsa-miR-34c-5p | MIMAT0000686 | 13.5 | 3.6 | 1.91 |
| hsa-miR-30e-3p | MIMAT0000693 | 29.8 | 12.7 | 1.23 |
| hsa-miR-375 | MIMAT0000728 | 62.7 | 27.3 | 1.20 |
| hsa-miR-148b-3p | MIMAT0000759 | 14.1 | 6.0 | 1.23 |
| hsa-miR-324-5p | MIMAT0000761 | 19.7 | 9.5 | 1.05 |
| hsa-miR-335-5p | MIMAT0000765 | 40.4 | 17.5 | 1.21 |
| hsa-miR-18b-5p | MIMAT0001412 | 19.5 | 8.5 | 1.20 |
| hsa-miR-20b-5p | MIMAT0001413 | 128.8 | 37.2 | 1.79 |
| hsa-miR-146b-5p | MIMAT0002809 | 168.7 | 67.3 | 1.33 |
| hsa-miR-519e-5p | MIMAT0002828 | 7.0 | 3.5 | 1.00 |
| hsa-miR-601 | MIMAT0003269 | 7.9 | 3.1 | 1.35 |
| hsa-miR-636 | MIMAT0003306 | 9.4 | 4.7 | 1.00 |
| hsa-miR-652-3p | MIMAT0003322 | 14.5 | 6.6 | 1.14 |
| hsa-miR-425-5p | MIMAT0003393 | 60.0 | 26.1 | 1.20 |
| hsa-miR-22-5p | MIMAT0004495 | 18.2 | 8.7 | 1.06 |
| hsa-miR-29b-2-5p | MIMAT0004515 | 9.0 | 3.5 | 1.36 |
| hsa-miR-374b-5p | MIMAT0004955 | 16.9 | 6.8 | 1.31 |
| hsa-miR-1261 | MIMAT0005913 | 14.2 | 7.0 | 1.02 |
| hsa-miR-664a-3p | MIMAT0005949 | 65.2 | 32.1 | 1.02 |
| hsa-miR-4317 | MIMAT0016872 | 56.8 | 27.2 | 1.06 |
| hsa-miR-23c | MIMAT0018000 | 70.1 | 28.4 | 1.30 |
| hsa-miR-374c-5p | MIMAT0018443 | 42.4 | 16.9 | 1.33 |
| hsa-miR-5196-3p | MIMAT0021129 | 9.2 | 4.5 | 1.03 |
| hsa-miR-664b-3p | MIMAT0022272 | 180.1 | 70.7 | 1.35 |
| hsa-miR-6073 | MIMAT0023698 | 20.1 | 6.8 | 1.56 |
| hsa-miR-6087 | MIMAT0023712 | 4297.4 | 1216.1 | 1.82 |
| hsa-miR-6823-3p | MIMAT0027547 | 7.2 | 2.6 | 1.47 |
| hsa-miR-203a-5p | MIMAT0031890 | 7.0 | 3.4 | 1.04 |
