# Supplementary material for: Unique circulating microRNAs in relation to EGFR mutation status in Japanese smoker male with lung adenocarcinoma
Source: Oncotarget. 2017 Sep 30;8(70):114685–97. doi: 10.18632/oncotarget.21425 (PMC5777724; doi:10.18632/oncotarget.21425)
Supplement: Supplementary file 3 [file oncotarget-08-114685-s003.docx]

Supplementary Table 2 miRNAs expressed in EGFR-mut more than 2-fold as compared with EGFR-wt by microarray.

| **Plasma** |  | **Global normalization（ratio）** | |  |
| --- | --- | --- | --- | --- |
| **Name** | **ID** | **wt** | **mut** | **Log2Ratio** |
| hsa-miR-103a-3p | MIMAT0000101 | - | 47.3 | 4.00 |
| hsa-miR-223-3p | MIMAT0000280 | - | 80.7 | 4.00 |
| hsa-miR-1246 | MIMAT0005898 | 67.5 | 623.4 | 3.21 |
| hsa-miR-122-5p | MIMAT0000421 | 11.6 | 110.2 | 3.25 |
| hsa-miR-451a | MIMAT0001631 | 78.5 | 749.7 | 3.26 |
| hsa-miR-1290 | MIMAT0005880 | 9.5 | 124.1 | 3.71 |
| hsa-miR-16-5p | MIMAT0000069 | 10.2 | 140.3 | 3.79 |
| hsa-miR-92b-3p | MIMAT0003218 | 41.6 | 166.7 | 2.00 |
| hsa-miR-6778-5p | MIMAT0027456 | 75.5 | 350.4 | 2.21 |
| hsa-miR-107 | MIMAT0000104 | 9.2 | 45.0 | 2.30 |
| hsa-miR-23b-3p | MIMAT0000418 | 16.1 | 93.1 | 2.53 |
| hsa-miR-23a-3p | MIMAT0000078 | 12.9 | 83.9 | 2.70 |
| hsa-miR-6073 | MIMAT0023698 | 18.0 | 53.3 | 1.57 |
| hsa-miR-191-5p | MIMAT0000440 | 22.6 | 65.2 | 1.53 |
| hsa-miR-4480 | MIMAT0019014 | 14.2 | 40.8 | 1.52 |
| hsa-miR-93-5p | MIMAT0000093 | 8.8 | 24.7 | 1.49 |
| hsa-miR-1273f | MIMAT0020601 | 32.1 | 90.0 | 1.49 |
| hsa-miR-4779 | MIMAT0019938 | 9.2 | 25.2 | 1.46 |
| hsa-miR-3682-3p | MIMAT0018110 | 13.4 | 35.9 | 1.42 |
| hsa-miR-1910-3p | MIMAT0026917 | 10.8 | 28.4 | 1.40 |
| hsa-miR-619-5p | MIMAT0026622 | 142.6 | 370.9 | 1.38 |
| hsa-miR-4648 | MIMAT0019710 | 29.9 | 76.8 | 1.36 |
| hsa-miR-494-3p | MIMAT0002816 | 9.0 | 23.1 | 1.36 |
| hsa-let-7a-5p | MIMAT0000062 | 8.0 | 20.4 | 1.35 |
| hsa-miR-6131 | MIMAT0024615 | 441.8 | 1126.9 | 1.35 |
| hsa-miR-19b-3p | MIMAT0000074 | 7.6 | 19.3 | 1.34 |
| hsa-miR-1285-3p | MIMAT0005876 | 17.5 | 43.4 | 1.31 |
| hsa-miR-3936 | MIMAT0018351 | 8.4 | 20.5 | 1.29 |
| hsa-miR-3135b | MIMAT0018985 | 214.9 | 518.9 | 1.27 |
| hsa-miR-92a-3p | MIMAT0000092 | 129.2 | 306.8 | 1.25 |
| hsa-miR-1972 | MIMAT0009447 | 12.2 | 28.0 | 1.20 |
| hsa-miR-3922-5p | MIMAT0019227 | 9.2 | 21.2 | 1.20 |
| hsa-miR-5585-3p | MIMAT0022286 | 45.9 | 104.9 | 1.19 |
| hsa-miR-3192-5p | MIMAT0015076 | 7.4 | 16.2 | 1.13 |
| hsa-miR-425-5p | MIMAT0003393 | 6.7 | 14.6 | 1.12 |
| hsa-miR-4436b-3p | MIMAT0019941 | 9.3 | 20.1 | 1.12 |
| hsa-miR-6838-5p | MIMAT0027578 | 7.8 | 17.0 | 1.12 |
| hsa-let-7c-5p | MIMAT0000064 | 10.8 | 23.1 | 1.10 |
| hsa-miR-4710 | MIMAT0019815 | 14.5 | 30.9 | 1.10 |
| hsa-miR-3619-3p | MIMAT0019219 | 358.8 | 764.2 | 1.09 |
| hsa-miR-612 | MIMAT0003280 | 9.9 | 21.1 | 1.09 |
| hsa-miR-3620-3p | MIMAT0018001 | 10.5 | 22.1 | 1.08 |
| hsa-miR-1273g-3p | MIMAT0022742 | 200.1 | 412.9 | 1.05 |
| hsa-miR-6890-5p | MIMAT0027680 | 20.3 | 40.9 | 1.01 |
| hsa-miR-4428 | MIMAT0018943 | 22.0 | 44.0 | 1.00 |
| hsa-miR-1273e | MIMAT0018079 | 28.1 | 84.0 | 1.58 |
| hsa-miR-1273a | MIMAT0005926 | 7.7 | 24.4 | 1.66 |
| hsa-miR-22-3p | MIMAT0000077 | 12.9 | 42.9 | 1.73 |
| hsa-miR-4732-5p | MIMAT0019855 | 30.8 | 107.5 | 1.80 |
| hsa-miR-15b-5p | MIMAT0000417 | 6.9 | 24.7 | 1.84 |
| hsa-miR-25-3p | MIMAT0000081 | 8.7 | 31.8 | 1.87 |
| **Tissue** | | | | |
| hsa-miR-505-3p | MIMAT0002876 | 2.9 | 12.5 | 2.12 |
| hsa-miR-513c-5p | MIMAT0005789 | 8.1 | 37.2 | 2.20 |
| hsa-miR-3678-3p | MIMAT0018103 | 55.2 | 255.9 | 2.21 |
| hsa-miR-4635 | MIMAT0019692 | 10.2 | 45.2 | 2.15 |
| hsa-miR-659-5p | MIMAT0022710 | 6.7 | 35.2 | 2.39 |
| hsa-miR-198 | MIMAT0000228 | 11.2 | 23.3 | 1.06 |
| hsa-miR-181a-3p | MIMAT0000270 | 4.4 | 10.3 | 1.21 |
| hsa-miR-127-3p | MIMAT0000446 | 3.9 | 9.4 | 1.29 |
| hsa-miR-184 | MIMAT0000454 | 3.2 | 7.1 | 1.14 |
| hsa-miR-485-5p | MIMAT0002175 | 6.7 | 13.5 | 1.01 |
| hsa-miR-494-3p | MIMAT0002816 | 792.1 | 2472.4 | 1.64 |
| hsa-miR-526b-3p | MIMAT0002836 | 5.5 | 18.8 | 1.78 |
| hsa-miR-564 | MIMAT0003228 | 36.3 | 79.8 | 1.14 |
| hsa-miR-615-3p | MIMAT0003283 | 19.4 | 39.3 | 1.02 |
| hsa-miR-625-5p | MIMAT0003294 | 2.9 | 7.1 | 1.29 |
| hsa-miR-628-3p | MIMAT0003297 | 3.9 | 9.6 | 1.29 |
| hsa-miR-654-5p | MIMAT0003330 | 3.0 | 10.0 | 1.74 |
| hsa-miR-542-5p | MIMAT0003340 | 9.2 | 22.5 | 1.29 |
| hsa-miR-363-5p | MIMAT0003385 | 18.6 | 71.5 | 1.95 |
| hsa-miR-767-5p | MIMAT0003882 | 4.3 | 13.7 | 1.66 |
| hsa-miR-765 | MIMAT0003945 | 57.4 | 162.9 | 1.50 |
| hsa-miR-129-1-3p | MIMAT0004548 | 3.5 | 8.5 | 1.29 |
| hsa-miR-938 | MIMAT0004981 | 4.2 | 11.2 | 1.44 |
| hsa-miR-513b-5p | MIMAT0005788 | 17.7 | 46.7 | 1.40 |
| hsa-miR-1293 | MIMAT0005883 | 7.7 | 16.3 | 1.08 |
| hsa-miR-365a-5p | MIMAT0009199 | 84.6 | 171.7 | 1.02 |
| hsa-miR-2277-3p | MIMAT0011777 | 4.3 | 10.4 | 1.26 |
| hsa-miR-3124-5p | MIMAT0014986 | 9.2 | 18.5 | 1.01 |
| hsa-miR-3130-3p | MIMAT0014994 | 3.2 | 8.6 | 1.43 |
| hsa-miR-1273c | MIMAT0015017 | 93.9 | 233.0 | 1.31 |
| hsa-miR-3150a-3p | MIMAT0015023 | 53.6 | 121.8 | 1.18 |
| hsa-miR-3074-3p | MIMAT0015027 | 3.2 | 6.6 | 1.04 |
| hsa-miR-3155a | MIMAT0015029 | 4.0 | 9.7 | 1.27 |
| hsa-miR-3161 | MIMAT0015035 | 31.5 | 94.2 | 1.58 |
| hsa-miR-4321 | MIMAT0016874 | 78.8 | 247.0 | 1.65 |
| hsa-miR-4265 | MIMAT0016891 | 6.9 | 22.7 | 1.71 |
| hsa-miR-3649 | MIMAT0018069 | 46.5 | 142.8 | 1.62 |
| hsa-miR-3692-5p | MIMAT0018121 | 5.5 | 11.1 | 1.01 |
| hsa-miR-3713 | MIMAT0018164 | 4.9 | 10.5 | 1.11 |
| hsa-miR-3714 | MIMAT0018165 | 74.0 | 164.9 | 1.16 |
| hsa-miR-3916 | MIMAT0018190 | 31.4 | 63.4 | 1.01 |
| hsa-miR-3150b-3p | MIMAT0018194 | 2.6 | 6.6 | 1.32 |
| hsa-miR-3934-5p | MIMAT0018349 | 51.7 | 122.5 | 1.25 |
| hsa-miR-3935 | MIMAT0018350 | 6.9 | 14.1 | 1.04 |
| hsa-miR-4435 | MIMAT0018951 | 99.4 | 213.8 | 1.10 |
| hsa-miR-4450 | MIMAT0018971 | 102.1 | 343.6 | 1.75 |
| hsa-miR-4462 | MIMAT0018986 | 73.8 | 183.4 | 1.31 |
| hsa-miR-4468 | MIMAT0018995 | 4.9 | 9.9 | 1.01 |
| hsa-miR-4470 | MIMAT0018997 | 13.3 | 33.3 | 1.33 |
| hsa-miR-4529-3p | MIMAT0019068 | 5.1 | 14.8 | 1.54 |
| hsa-miR-3177-5p | MIMAT0019215 | 3.1 | 7.3 | 1.25 |
| hsa-miR-4644 | MIMAT0019704 | 4.9 | 11.7 | 1.24 |
| hsa-miR-4653-3p | MIMAT0019719 | 17.1 | 42.2 | 1.30 |
| hsa-miR-4684-3p | MIMAT0019770 | 10.8 | 41.3 | 1.93 |
| hsa-miR-4688 | MIMAT0019777 | 259.9 | 647.2 | 1.32 |
| hsa-miR-4691-5p | MIMAT0019781 | 11.2 | 24.7 | 1.13 |
| hsa-miR-4694-3p | MIMAT0019787 | 5.4 | 12.8 | 1.24 |
| hsa-miR-4756-5p | MIMAT0019899 | 92.6 | 199.8 | 1.11 |
| hsa-miR-4761-3p | MIMAT0019909 | 3.2 | 6.9 | 1.09 |
| hsa-miR-5003-3p | MIMAT0021026 | 2.9 | 6.0 | 1.04 |
| hsa-miR-5006-5p | MIMAT0021033 | 57.2 | 130.7 | 1.19 |
| hsa-miR-652-5p | MIMAT0022709 | 278.3 | 623.2 | 1.16 |
| hsa-miR-766-5p | MIMAT0022714 | 6.0 | 13.3 | 1.15 |
| hsa-miR-1236-5p | MIMAT0022945 | 17.3 | 42.4 | 1.29 |
| hsa-miR-6075 | MIMAT0023700 | 1349.1 | 2825.7 | 1.07 |
| hsa-miR-6083 | MIMAT0023708 | 2.7 | 7.3 | 1.41 |
| hsa-miR-6084 | MIMAT0023709 | 3.0 | 6.8 | 1.16 |
| hsa-miR-6129 | MIMAT0024613 | 6.2 | 15.7 | 1.34 |
| hsa-miR-6165 | MIMAT0024782 | 78.5 | 167.3 | 1.09 |
| hsa-miR-6501-5p | MIMAT0025458 | 7.6 | 16.3 | 1.09 |
| hsa-miR-6715b-5p | MIMAT0025842 | 29.3 | 76.2 | 1.38 |
| hsa-miR-520g-5p | MIMAT0026611 | 7.2 | 15.3 | 1.08 |
| hsa-miR-1266-3p | MIMAT0026742 | 9.0 | 27.2 | 1.60 |
| hsa-miR-6731-5p | MIMAT0027363 | 154.0 | 455.8 | 1.57 |
| hsa-miR-6743-5p | MIMAT0027387 | 856.7 | 2189.4 | 1.35 |
| hsa-miR-6769b-5p | MIMAT0027620 | 140.2 | 326.2 | 1.22 |
| hsa-miR-6864-5p | MIMAT0027628 | 5.8 | 18.7 | 1.69 |
| hsa-miR-6875-3p | MIMAT0027651 | 17.0 | 40.1 | 1.23 |
| hsa-miR-6877-5p | MIMAT0027654 | 236.1 | 540.4 | 1.19 |
| hsa-miR-6882-3p | MIMAT0027665 | 2.8 | 6.6 | 1.23 |
| hsa-miR-6893-5p | MIMAT0027686 | 306.4 | 660.6 | 1.11 |
| hsa-miR-6895-5p | MIMAT0027690 | 289.9 | 902.9 | 1.64 |
| hsa-miR-7113-5p | MIMAT0028123 | 7.8 | 16.2 | 1.04 |
| hsa-miR-7150 | MIMAT0028211 | 1089.9 | 2773.5 | 1.35 |
| hsa-miR-7154-3p | MIMAT0028219 | 22.2 | 52.1 | 1.23 |
| hsa-miR-7641 | MIMAT0029782 | 2150.0 | 4622.2 | 1.10 |
| hsa-miR-4485-5p | MIMAT0032116 | 279.2 | 577.6 | 1.05 |
| hsa-miR-7854-3p | MIMAT0030429 | 26.9 | 54.4 | 1.02 |
