# Supplementary material for: Unique circulating microRNAs in relation to EGFR mutation status in Japanese smoker male with lung adenocarcinoma
Source: Oncotarget. 2017 Sep 30;8(70):114685–97. doi: 10.18632/oncotarget.21425 (PMC5777724; doi:10.18632/oncotarget.21425)
Supplement: Supplementary file 4 [file oncotarget-08-114685-s004.docx]

Supplementary Table 3 Comparison of 17 miRNAs expression between current- and former-smoker.

| Smokers |  | All stage (68) | | |  | Stage I (45) | | |  | Stage I+II (58) | | |
| --- | --- | --- | --- | --- | --- | --- | --- | --- | --- | --- | --- | --- |
| miRNA |  | Current | Former | *P*-value |  | Current | Former | *P*-value |  | Current | Former | *P*-value |
| miR-16-5p |  | 2.52 | 1.52 | 0.12 |  | 2.35 | 1.18 | **0.03** |  | 2.52 | 1.4 | 0.11 |
| miR-23a-3p |  | 7.45 | 8.77 | 0.08 |  | 8.64 | 8.94 | 0.43 |  | 7.51 | 8.85 | 0.19 |
| miR-92b-3p |  | 17.06 | 19.99 | 0.19 |  | 16.88 | 17.07 | 0.65 |  | 16.93 | 19.66 | 0.25 |
| miR-103a-3p |  | 7.17 | 7.2 | 0.40 |  | 7.17 | 6.68 | 0.17 |  | 7.24 | 7.2 | 0.33 |
| miR-122-5p |  | 7.91 | 7.28 | 0.48 |  | 7.38 | 6.74 | 0.84 |  | 7.69 | 6.98 | 0.51 |
| miR-192-5p |  | 8.41 | 8.81 | 0.42 |  | 8.34 | 8.57 | 0.41 |  | 8.34 | 8.81 | 0.44 |
| miR-194-5p |  | 12.45 | 11.99 | 0.53 |  | 13.43 | 11.99 | 0.36 |  | 13.02 | 11.99 | 0.41 |
| miR-223-3p |  | 6.53 | 5.75 | 0.32 |  | 6.1 | 5.51 | 0.21 |  | 6.53 | 5.57 | 0.28 |
| miR-346 |  | 15.02 | 14.09 | 0.14 |  | 14.83 | 13.44 | 0.10 |  | 14.83 | 13.68 | 0.14 |
| miR-451a |  | 0.59 | 0.26 | 0.22 |  | 0.59 | -0.64 | **0.05** |  | 0.93 | 0.09 | 0.12 |
| miR-619-5p |  | 13.27 | 13.03 | 0.41 |  | 13.33 | 12.68 | 0.36 |  | 13.32 | 13.03 | 0.61 |
| miR-1246 |  | 7.66 | 7.18 | 0.88 |  | 7.84 | 7.07 | 0.64 |  | 7.84 | 7.09 | 0.86 |
| miR-1290 |  | 16.91 | 17.51 | 0.90 |  | 16.1 | 15.36 | 0.71 |  | 17.03 | 16.45 | 0.63 |
| miR-4704-3p |  | 20.74 | 16.95 | 0.14 |  | 21.91 | 15.14 | 0.06 |  | 20.81 | 15.91 | 0.06 |
| miR-4732-5p |  | 14.6 | 14.83 | 0.74 |  | 18.23 | 14.39 | 0.56 |  | 15.18 | 14.75 | 0.99 |
| miR-6765-3p |  | 23.79 | 21.35 | 0.31 |  | 23.56 | 22.43 | 0.40 |  | 23.79 | 21.21 | 0.19 |
| miR-6778-5p |  | 14.45 | 13.46 | 0.11 |  | 14.45 | 13.33 | 0.11 |  | 14.46 | 13.46 | 0.06 |

The expression levels of miRNAs were calculated by using the delta Ct method (dCt = Ct _test_ - Ct _ath-miR-159a_). Each median value is shown. The *P*-values were determined by Mann-Whitney U test. Bold: P<0.05
